# Preparation and Properties of Novel Thermoplastic Vulcanizate Based on Bio-Based Polyester/Polylactic Acid, and Its Application in 3D Printing

**DOI:** 10.3390/polym9120694

**Published:** 2017-12-09

**Authors:** Yu Gao, Yan Li, Xiaoran Hu, Weidong Wu, Zhao Wang, Runguo Wang, Liqun Zhang

**Affiliations:** 1 Key Laboratory of Beijing City for Preparation and Processing of Novel Polymer Materials, Beijing University of Chemical Technology, Beijing 100029, China; 2015200565@grad.buct.edu.cn (Y.G.) ; 2014200540@grad.buct.edu.cn (Y.L.); 201440099@mail.buct.edu.cn (X.H.); wuweidong@buct.edu.cn (W.W.); wangzhao@buct.edu.cn (Z.W.); 2 State Key Laboratory of Organic-Inorganic Composites, Beijing University of Chemical Technology, Beijing 100029, China; 3 Beijing Laboratory of Biomedical Materials, Beijing University of Chemical Technology, Beijing 100029, China

**Keywords:** polyester, bio-based elastomer, polylactic acid, thermoplastic vulcanizate

## Abstract

Thermoplastic vulcanizate (TPV) combines the high elasticity of elastomers and excellent processability of thermoplastics. Novel bio-based TPV based on poly (lactide) (PLA) and poly (1,4-butanediol/2,3-butanediol/succinate/itaconic acid) (PBBSI) were prepared in this research. PBBSI copolyesters were synthesized by melting polycondensation, and the molecular weights, chemical structures and compositions of the copolyesters were characterized by GPC, NMR and FTIR. Bio-based 2,3-butanediol was successfully incorporated to depress the crystallization behavior of the PBBSI copolyester. With an increase of 2,3-butanediol content, the PBBSI copolyester transformed from a rigid plastic to a soft elastomer. Furthermore, the obtained TPV has good elasticity and rheological properties, which means it can be applied as a 3D-printing material.

## 1. Introduction

Thermoplastic vulcanizate (TPV) attracts considerable attention owing to its excellent performance and extensive applications in replacing thermoset elastomers, such as in automotive profiles, window gaskets, tubes, seals, electrical wires and cables [1,2]. They are soft and flexible materials with relatively high tensile strength and elongation at break, and fair elasticity. TPVs can be processed by common melt-processing techniques, like blow molding, injection molding and extrusion, while production scrap can be recycled easily [3]. It possesses the fine resilience of conventional vulcanized elastomers, along with the good processability and easy recyclability of thermoplastics [4,5,6]. TPV is produced by dynamic vulcanization, making a high content of crosslinked elastomer as the dispersed phase and a low content of thermoplastic as the continuous phase [7]. The crosslinked elastomer particles ensure the elasticity by means of intense mechanical shearing, breaking up into large amounts of micron-sized particles, dispersed in a continuous thermoplastic matrix [8,9,10]. Most commercial TPVs consist of ethylene–propylene–diene terpolymer (EPDM) as the elastomer phase and semi-crystalline, isotactic polypropylene (iPP) as the thermoplastic phase. The specific combination of EPDM and iPP results in an excellent resistance against oxidation and thermal degradation, as well as high temperature resistance. They usually were petroleum-based materials [1,11,12], leading to environmental pollution and accelerated consumption of oil resources.

The environmental issues have always been the focus of attention, and in order to reduce environmental pollution and consumption of petroleum energy, it is an important solution to develop various bio-based materials including starch-based polymers [13], polylactic acid (PLA) [14], 1,3-propanediol-based polymers [15,16], polyhydroxyalkanoates (PHAs) [17], polybutylene succinate (PBS) [18] and so on. Until now, PLA is believed to be the most successful bio-based polymer with a great potential market [19]. However, there are few commercial bio-based elastomers. Bio-based elastomers are synthetized with monomers sourced from renewable raw materials by biological transformation. Some bio-based monomers have been used for the synthesis of polyester-type plastics, such as butanediol, isosorbide, bicyclic dicarboxylic acid, galactose and furan-2,5-dicarboxylic acid. 2,3-butanediol is a promising and valuable chemical that can be used in various areas as a liquid fuel and a platform monomer [20], and it can be produced by conventional fermentation from various bioresources such as xylose, sugar cane molasses, corn and glucose [21]. It has pendent groups, which can effectively inhibit the crystallization of the polyester elastomer. Performance of the polyester changes correspondingly with a change of 2,3-butanediol content; these properties include molecular weight, molecular weight distribution, crystallization behavior and mechanical properties. What is more, Yi Jiang et al. [22] used bio-based dimethyl itaconate, 1,4-butanediol and various diacid ethyl compounds, via Candida antarctica lipase B (CALB)-catalyzed polycondensation, to produce various bio-based saturated aliphatic polyesters and itaconate-based unsaturated aliphatic polyesters. Christian Hoffmann et al. also used a CALB-catalyzed polycondensation reaction to provide a polyester [23]. This was an improvement in the synthesis of polyesters.

Nowadays, 3D printing plays a crucial role in combining scientific discovery with material innovation, promoting the development and progress of the manufacturing industry [24]. It consists of many types of printing modes; for instance: fused deposition modeling (FDM), selective laser sintering (SLS), direct metal laser sintering (DMLS), stereolithography (STL) and laminated object manufacturing [25]. The materials of 3D printing in FDM mode are thermoplastics, eutectic system metals and edible materials. Thi Nga Tran et al. [26] used cocoa-shell waste bio-filaments in 3D printing, which was a new material for this application, and his work has a great significance in the development of environmental protection. PLA-based materials have been widely used in 3D printing; for example, Christopher M. O’Brien et al. used polylactic acid to print scaffolds for organ supports [27]. Park et al. fabricated scaffolds of tooth dentin–ligament–bone complexes by 3D printing [28,29]. They all studied 3D-printing technology for medical stent structures, which require materials to be biodegradable, and the structures play a supporting role, requiring higher material hardness. However, PLA also has some drawbacks, for example, it is not suitable for printing products with flexible requirements owing to its high hardness and high toughness. Therefore, elastic materials are also highly needed in 3D printing for not only medical applications but also other fields. Lee et al. developed a skin biofabrication method using a 3D bioprinter with different dispensers independently operated by electromechanical valves and mounted on three-axis robotic stages [30,31]. What is more, Kang [32] and Hu [33,34,35] have prepared different types of polyester elastomers with versatile applications using bio-based monomers like lactic acid, sebacic acid, itaconic acid, 1,4-butanediol (BDO) and so on. We want to blend our polyesters with PLA through a series of processing methods, getting poly (1,4-butanediol/2,3-butanediol/succinic acid/itaconate) (PBBSI) elastomers/PLA TPVs which have the good elasticity of PBBSI as a dispersed phase and also have the excellent processability of PLA as a continuous phase.

The aim of this study is to design and synthesize novel bio-based poly (1,4-butanediol/2,3-butanediol/succinic acid/itaconate) (PBBSI) copolyesters with different 2,3-butanediol contents, and then prepare 3D-printable TPVs based on PBBSI and PLA. The synthesized copolyesters varied from rigid plastics to flexible elastomers with different 2,3-butanediol contents. The effects of composition on the microstructures, thermal properties, mechanical properties, rheological properties and versatile applications were investigated.

## 2. Materials and Methods

### 2.1. Materials

2,3-Butanediol (2,3-BDO, purity 97.8%) was prepared in our laboratory according to the procedure given in previous work [36,37]. Succinic acid (SuA), 1,4-butanediol (1,4-BDO purity 97.8%), itaconic acid (IA purity 98%), tetrabutyltitanate (TBT) and dicumyl peroxide (DCP) were purchased from Alfa Aesar Chemical Inc. (Ward Hill, MA, USA). Chloroform and methanol were supplied by Beijing Chemical Works (Beijing, China). Polylactide (PLA 2003D) was supplied by America Natureworks (Blair, NE, USA).

### 2.2. Synthesis of PBBSI Copolyesters

**Scheme 1:**
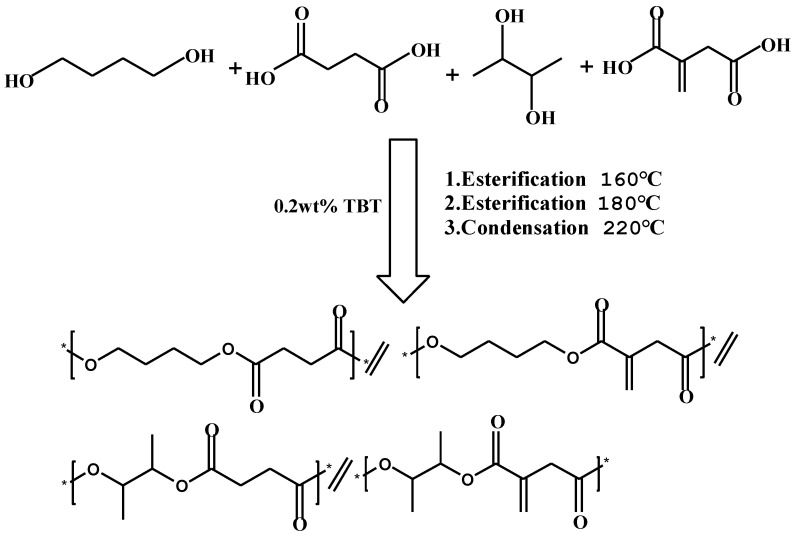
The reaction formula of PBBSI copolyesters.

The synthesis of PBBSI includes a three-stage polymerization process, as shown in Scheme 1. The molar ratio of hydroxyl/carboxyl was fixed at 1.1:1. The molar ratio of 2,3-BDO relative to the total alcohol amount (2,3-BDO and 1,4-BDO) varied from 50 to 90 mol %. For example, in the preparation of PBBSI-70, calculated amounts of 2,3-BDO (21.483 g, 0.239 mol), SuA (32.922 g, 0.279 mol), 1,4-BDO (9.207 g, 0.102 mol) and IA (4.030 g, 0.031 mol) were heated at 160 °C for 3 h and then at 180 °C for 2 h in a 100 mL three-neck flask equipped with a mechanical stirrer under nitrogen atmosphere. The polymerization was performed at 220 °C for 6 to 8 h under reduced pressure (< 300 Pa) with TBT (0.2 wt % relative to all chemicals) as the catalyst until the Weisenberg effect was observed. The polymerization was carried out in the presence of a tri-(4-hydroxy-TEMPO) phosphate (0.0676 g, 0.1 wt % relative to all chemicals) as an antioxidant and an inhibitor. The obtained copolyester was dissolved in chloroform and precipitated in excess methanol to remove any unreacted monomers and remaining oligomers. Finally, the copolyester was collected by filtration, extensively washed with methanol, and dried under vacuum at 60 °C. The unsaturated copolyester containing 70 mol % of 2,3-BDO relative to all chemical amounts was denoted as PBBSI-70. PLA was synthesized by direct polycondensation from lactic acid monomer. Before the preparation of TPV, the PBBSI and PLA were dried under vacuum at 40 °C for 24 h to remove the solvent.

### 2.3. Preparation of PBBSI/PLA TPV

The PBBSI and PLA were dried in a vacuum oven at 60 °C for 24 h prior to use. PBBSI/PLA TPV was prepared by melt-blending PBBSI with PLA at 60:40, for 10 min in a Haake mixer at 160 °C and a rotational speed of 80 rpm. The PBBSI/PLA premix and DCP were mixed to obtain a blend on a 6-inch two-roll mill at room temperature. Then, the PBBSI/PLA blend was dynamically crosslinked for 8 min at 160 °C at a rotational speed of 80 rpm in the Haake mixer. The dynamically crosslinked TPV was hot-pressed at 180 °C to form 1-mm-thick sheets.

### 2.4. Characterization and Measurements

The GPC (Water, Milford, MA, USA) used tetrahydrofuran (THF) as solvent to characterise number-average molecular weight and dispersion index on a Waters Breeze instrument equipped with three water columns (Styragel HT3_HT5_HT6E) using tetrahydrofuran (THF) as the eluent (1 mL/min) and a Waters 2410 refractive index detector (Water, Milford, MA, USA), and the standard polystyrene flow was used for correction. The FTIR spectra of the copolyesters were recorded on a Bruker Tensor 27 spectrometer (Bruker, Rheinstetten, Germany)). The spectra were obtained in the wavenumber range 4000 to 500 cm^−1^ at a resolution of 4 cm^−1^. The peaks of the spectra were normalized at 1731 cm^−1^. ^1^H NMR spectra of the polyester were obtained on a Bruker AV600 spectrometer (Bruker, Rheinstetten, Germany). The solvent for the measurement was CDCl_3_. The peaks of the spectra were normalized at 2.64 ppm. A STARe system TGA apparatus (Mettler-Toledo International Inc.,Greifensee, Switzerland) was applied to test the thermal stability of the samples. The polyester weighing 10 mg was heated from 25 to 800 °C at a heating rate of 10 °C min^−1^ under a nitrogen flow. Differential scanning calorimetry (DSC) measurements were performed with a Mettler-Toledo DSC instrument (Mettler-Toledo International Inc., Greifensee, Switzerland) under nitrogen. To remove any previous thermal history, a sample was heated to 200 °C at 10 °C·min^−1^ and kept isothermal for 5 min. Then, it was cooled to −100 °C at 10 °C·min^−1^ and reheated up to 200 °C at 10 °C·min^−1^. X-ray diffraction (XRD) measurements were carried out on a D/Max2500 VB2t/PC X-ray diffractometer (Rigaku, Tokyo, Japan) with a Cu target radiation in a 2θ range of 5–50° at an angular resolution of 0.5°. A cryo-ultramicrotome (Leica EM UC7, Rheinstetten, Germany) equipped with a glass knife at −100 °C was used for the atom force microscopy (AFM), then observed under a Nanoscope IIIa peak force QNM AFM (Bruker, Rheinstetten, Germany). Tensile tests equipped with a 500 N load-cell were carried out on a CMT 4104 Electrical Tensile Instrument (Shenzhen SANS Test Machine Co., Ltd., Shenzhen, China). Dumbbell-shaped samples (1 mm thick and 4 mm wide) were measured at 25 °C and a crosshead speed of 200 mm·min^−1^ according to ASTM D412. For each measurement, five samples were tested and the average was taken. Dynamic mechanical thermal analysis was carried out with a V Dynamic Mechanical Thermal Analyzer (Rheometric Scientific Co, Limonest, France) with a tension mode at 1 Hz and 3 ℃/min from −80 to 80 ℃. Testing of rheological properties was carried out on a Mars III Rotational Rheometer (Malvern instruments limited, Malvern, UK) at the temperatures of 190, 200 and 210 °C, using the weight of 30 to 40 g. TPV was tested at 190 °C under the load of 2.15 kg by a melt-flow indexer (Jin Jian Testing Instrument Co., Ltd., Chengde, China). Hardness characterization was made by Rockwell hardness tester (Bareiss Prufgeratebau GmbH, Oberdischingen, Germany) using a 6 mm plate-shaped material in height gauge, the sample was tested three times and we took the average. For the 3D-printing process, the TPV was printed at 170 °C, using a Wiiboox ONE 3D printer (Wiiboox Technology Co Ltd., Nanjing, China).

## 3. Results and Discussion

### 3.1. Synthesis and Characterization of PBBSI Copolyesters 

Through a three-step polymerization, the Weissenberg effect was observed, and PBBSI, with a weight fraction of 2,3-BDO ranging from 40% to 80%, could be obtained. Owing to the steric effect of methyl groups of 2,3-BDO, the molecular weight of PBBSI gradually decreased with an increase of 2,3-BDO content. The reaction time also increased with an increase of 2,3-BDO content, and the Weissenberg effect was absent when the 2,3-BDO content was increased to 100%. The number-average molecular weight (*M*_n_) of PBBSI ranged from 23,000 to 39,000, and polydispersities (PDIs) varied from 3.2 to 5.0, as shown in Table 1.

Number-average molecular weights (*M*_n_) and polydispersity indices (PDI) determined by GPC in THF against PS(Polystyrene:the standard sample) standards.

**Figure 1:**
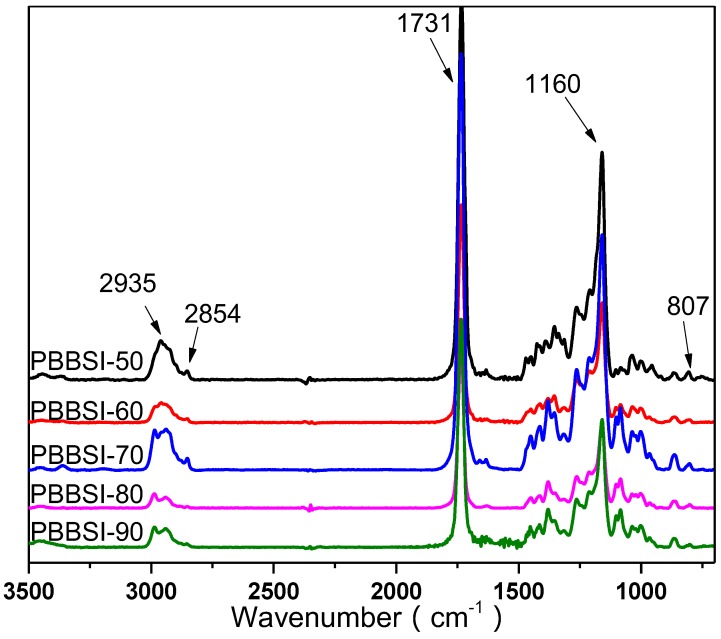
FTIR spectra of PBBSI copolyesters.

Fourier-transform infrared spectroscopy (FTIR) was used to characterize the functional groups of PBBSI copolyesters, shown in Figure 1. All spectra exhibit similar absorptions. The absorptions of 2935 and 2854 cm^−1^ were the symmetric and the asymmetric stretching vibrations of methylene (−CH_2_), respectively. The absorption of 1731 cm^−1^ indicated the stretching vibration of the carbonyl group (C=O) in the ester, and the stretching vibration peak of the ester bond was shown in the absorption of 1163 cm^−1^. These two peaks demonstrated the presence of ester bonds in the PBBSI copolyester.

**Figure 2:**
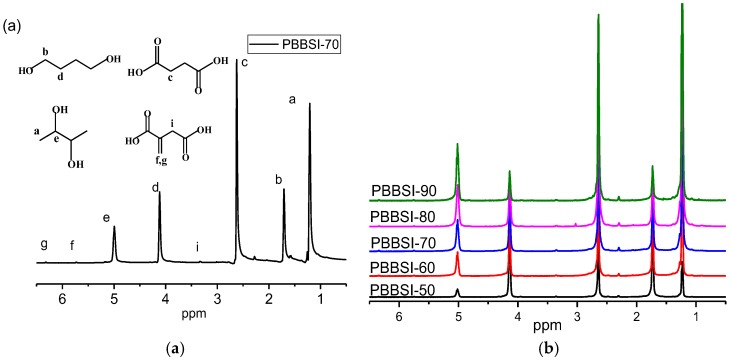
(**a**,**b**) ^1^H NMR spectra of PBBSI copolyesters.

^1^H NMR spectra of PBBSI copolyesters are shown in Figure 2b, and a representative ^1^H NMR spectrum of PBBSI-70 is shown in Figure 2a. The chemical shift of methylene groups in succinic acid –CH_2_–COO– was found at 2.64 ppm. The proton signals of –O–CH_2_–CH_2_– in 1,4-butanediol were at 4.15 and 1.73 ppm. The peaks at 1.23 and 5.01 ppm clearly show the methyne and the side methyl group of 2,3-BDO, respectively, indicating the existence of 2,3-BDO units in the obtained copolyester. The chemical shifts at 6.35, 5.73 and 3.36 ppm showed –CO–C (=CH_2_)–CH_2_– pertaining to the pendent alkene group and the methylene group in IA. ^1^H NMR spectrum of PBBSI copolyesters with different contents of 2,3-BDO is shown in Figure 2b. The proton peaks of each component with different contents of 2,3-BDO are similar. Observed from the figure, the intensity increased significantly with an increase of the ratio of 2,3-BDO, showing that the chemical composition of the polymer can be adjusted by regulating the ratio of 2,3-BDO. Calculating the area of the hydrogen proton peaks in the three kinds of monomer reveals the actual polyester chemical composition, as shown in Table 2. From the table, we can see that the actual composition ratio of 2,3-BDO in the PBBSI copolyester was slightly lower than the initial reaction ratio. We used suspended double bonds to characterize the content of IA. Then, the actual composition ratio of IA is only 40% of the feed values, and this is because a suspended double bond of IA leads to high steric hindrance, making IA difficult to be introduced.

### 3.2. Thermal and Crystalline Properties of PBBSI Copolyesters 

**Figure 3:**
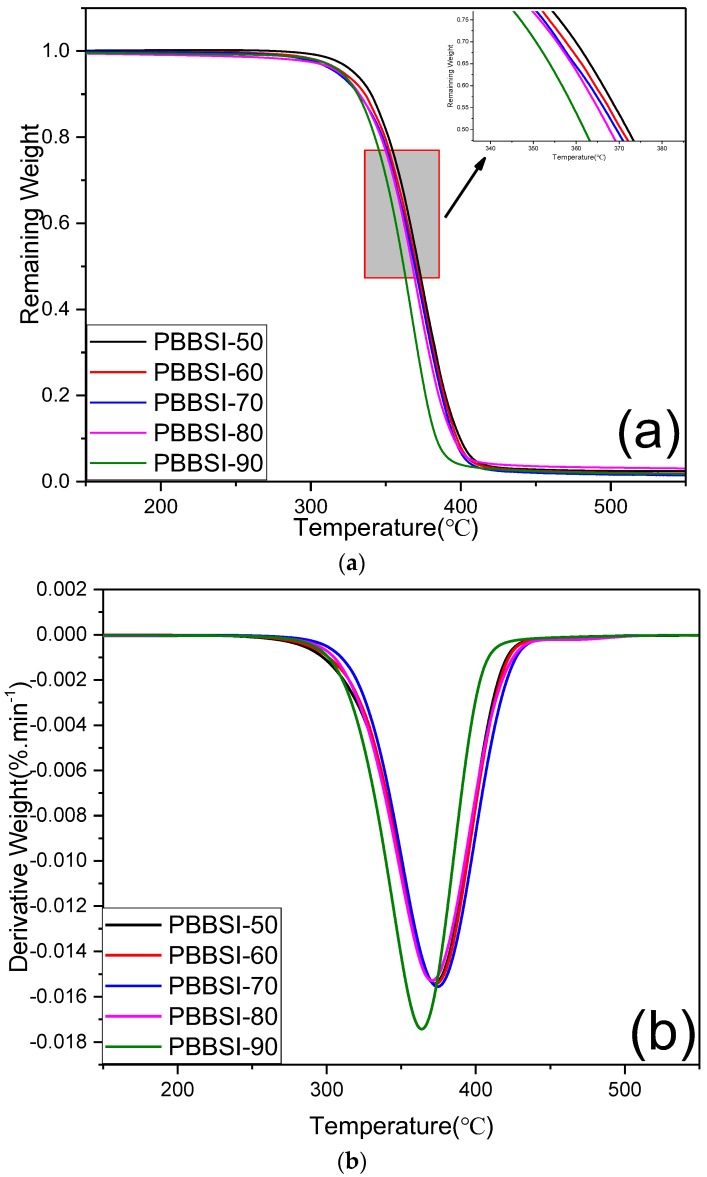
(**a**) TGA traces of the PBBSI copolyesters; (**b**) derivative curves of PBBSI copolyesters.

The thermogravimetric analysis (TGA) was conducted to characterize the thermal stability of the PBBSI copolyesters, as shown in Figure 3. It was conducted under a nitrogen atmosphere and the temperature ranged from 100 to 550 °C. The process was a one-step degradation, which started to decompose at 300 °C. The decomposition temperature gradually decreased with an increase of 2,3-BDO content, which suggests that the introduction of 2,3-BDO into the macromolecular chain weakened the thermal stability of the PBBSI copolyesters. Most of the decomposition process was distributed between 300 °C and 400 °C, leaving at least 3% in the final residue at 600 °C. We analyzed the temperatures at 5% weight loss (*T*_d_,_5%_) and at the maximum degradation rate (*T*_d,max_) for all the copolyesters, getting Table 3. *T*_d,5%_ and *T*_d,max_ decreased as the 2,3-BDO content increased, the same as the variation tendency of the initial decomposition temperature. As the content of 2,3-BDO increased, polymerization became more and more difficult because of its low reactivity. When it reached 90% of the total mass of alcohols, the polymerization time became longer, and many oligomers were produced in the system. Oligomers have poor thermal stability, and PBBSI-90 would have a low decomposition temperature as a whole, which caused a gap between PBBSI-90 and the rest.

**Figure 4:**
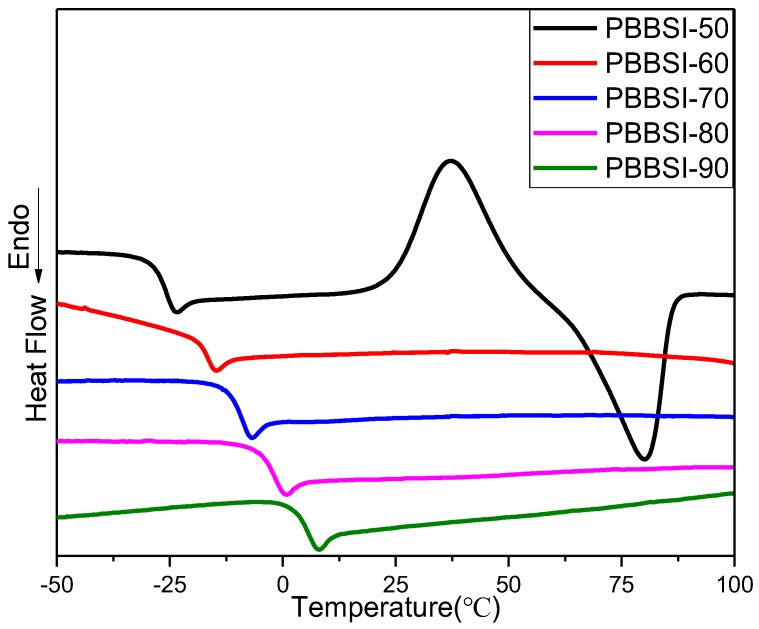
DSC curves of neat PBBSI copolyesters.

Differential scanning calorimetry (DSC) was conducted to investigate the thermal behaviors of PBBSI copolyesters. The cooling DSC curves of the PBBSI copolyesters can be observed in Figure 4. There was only one glass transition temperature (*T*_g_) of all the copolyesters in the cooling process. It ranged from −26 to 5 °C, and had a steady increase as the 2,3-BDO content increased, owing to the side methyl group of 2,3-BDO restricting the mobility of the macromolecular chains. What is more, as the 2,3-BDO content increased, PBBSI copolyesters changed from plastics to flexible elastomers at room temperature. Seen from Figure 4, there were sharp and intense crystallization peaks at temperatures in the range of −4 to 43 °C, proving that the PBBSI copolyester with 50 mol % 2,3-BDO content was semicrystalline. To observe the curve of the PBBSI-50 copolyester, the crystallization temperature (*T*_c_) was about 36 °C and the melting temperature (*T*_m_) was around 80 °C. However, no crystallization peaks can be observed in the graph of PBBSI-60, because all the samples were cooling at a rate of 10 °C/min^−1^ so there was not enough time for PBBSI copolyesters to crystallize.

**Figure 5:**
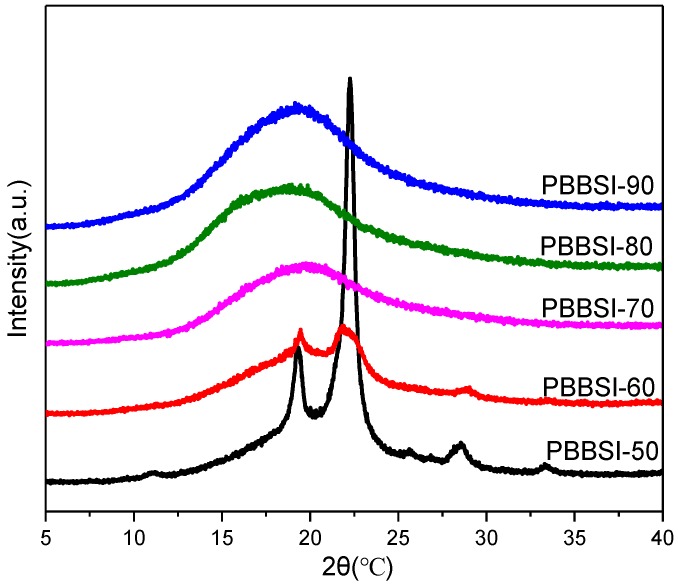
WAXD patterns of PBBSI copolyesters.

The wide-angle X-ray diffraction (WAXD) patterns are shown in Figure 5. There were two sharp diffraction peaks at 19.5° and 22.4° in the curves of PBBSI-50 and PBBSI-60, respectively, and peak height decreased as 2,3-BDO increased. The peak was absent in the curves of PBBSI-70, PBBSI-80 and PBBSI-90. This can be explained by the introduction of 2,3-BDO, which restricted the mobility of the macromolecular chains and inhibited the crystallization.

### 3.3. Mechanical Properties of PBBSI Copolyesters 

Stress–strain curves of PBBSI copolyesters are displayed in Figure S2 and Table S1 (see Supplementary Materials). Among the copolyesters, PBBSI-50 showed distinct yield points and typical plastic deformation, and it had a significantly higher tensile strength compared with other copolyesters. Samples from PBBSI-60 to PBBSI-90 exhibited obvious elastic deformation, and the tensile strength increased with an increase of 2,3-BDO content. The differences in the stress–strain curves proved that the PBBSI copolyesters transform from rigid plastics to flexible elastomers with increasing 2,3-BDO content, which is consistent with the results of XRD. Therefore, we can adjust the 2,3-BDO content in the copolymerization process to get a series of PBBSI copolyesters with tunable properties.

**Figure 6:**
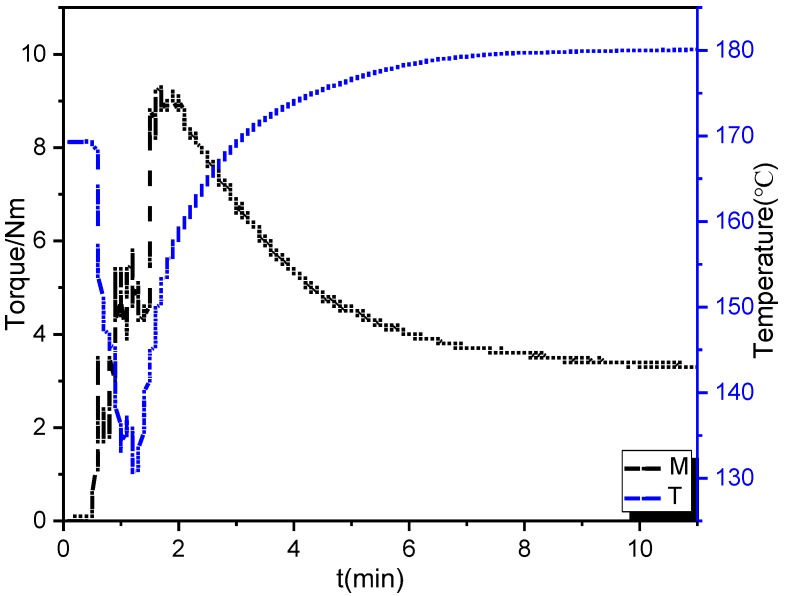
Dynamical vulcanization curves of PBBSI copolyesters.

Dynamic vulcanization is the process of melt-blending of the elastomer and thermoplastic polymer in a high-temperature and high-shear mixer; the elastomer was vulcanized in the presence of a crosslinking agent, obtaining a micron-sized vulcanized elastomer phase which was uniformly dispersed in the resin and very stable in processing. The vulcanization curve of PBBSI/PLA is shown in Figure 6. As the temperature increased, the torque increased to the highest point, and then, as the elastomer was rapidly vulcanized, the curve started to decrease into a steady state. At this time, the phase inversion had been completed, and the dynamic vulcanization ended.

### 3.4. Morphology of PBBSI/PLA TPV

The morphology of multiphase polymer blends has a great effect on their mechanical properties. AFM was used to identify the phase structure of the dynamically vulcanized PBBSI/PLA TPV, as shown in Figure S3 (see Supplementary Materials). A typical sea-island structure can be observed in the TPV; the dark part in the phase-angle images is the PLA phase, while the bright region corresponds to the PBBSI phase. According to the atomic force phase diagram, there was a good dispersion between the dark and bright parts. In other words, PBBSI particles are dispersed evenly in the continuous phase of the plastic, in the size of several microns.

### 3.5. Thermal and Crystalline Properties of PBBSI/PLA TPV

**Figure 7:**
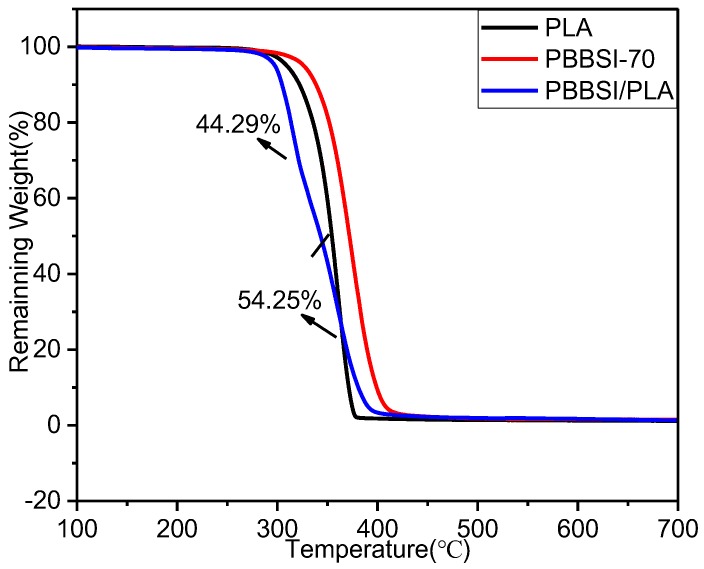
TGA traces of the PBBSI/PLA TPV and its individual components.

The thermal stability of the PBBSI/PLA TPV was studied by TGA, and the comparison of thermal stability curves with neat PLA and the PBBSI-70 copolyester is shown in Figure 7. A two-stage thermal decomposition was seen in the curve of PBBSI/PLA TPV, where the first stage was at about 285 °C, and the loss-weight was 44.29%, consistent with the decomposition of PLA. Then, when the temperature increased to 344 °C, the second stage appeared and the weight continued to decrease by 54.25%. As shown in Figure 7, the second stage of the PBBSI/PLA curve also corresponded with the thermal stability curve of PBBSI. The thermal stability of PBBSI was superior to PLA.

**Figure 8:**
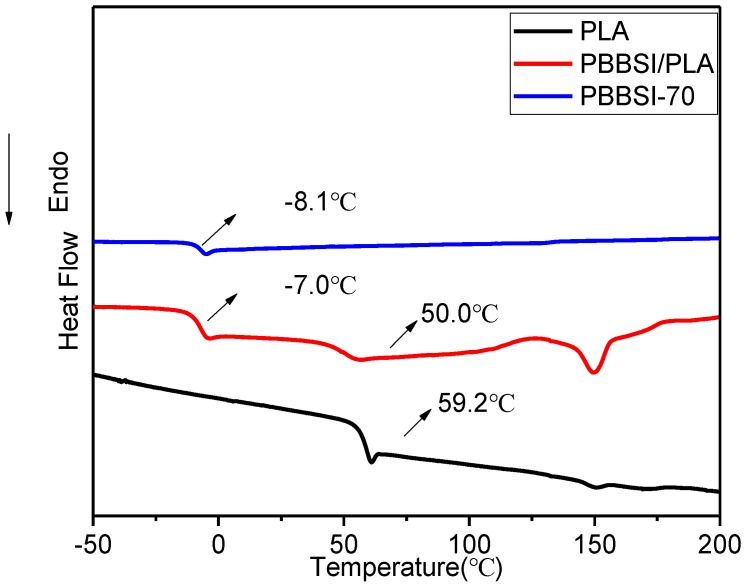
DSC curves of the PBBSI/PLA TPV and its individual components.

Seen from Figure 8, the DSC curve of PLA had one glass transition temperature at −8.1 °C, and that of PBBSI-70 also had only one glass transition temperature at 59.2 °C. There were two glass transition temperatures in the curve of PBBSI/PLA, which were at −7.0 and 50.0 °C; being between the *T*_g_ of PLA and the *T*_g_ of PBBSI, this indicated that the TPV was phase-separated during cooling. What is more, we can see the that two glass transition temperatures of the PBBSI/PLA TPV were closer to each other compared to the glass transition of PLA and PBBSI-70, showing the good compatibility of PLA and PBBSI in the TPV. The good compatibility of the two phases can be further testified by atomic force microscopy.

### 3.6. Mechanical Properties of the PBBSI/PLA TPV

**Figure 9:**
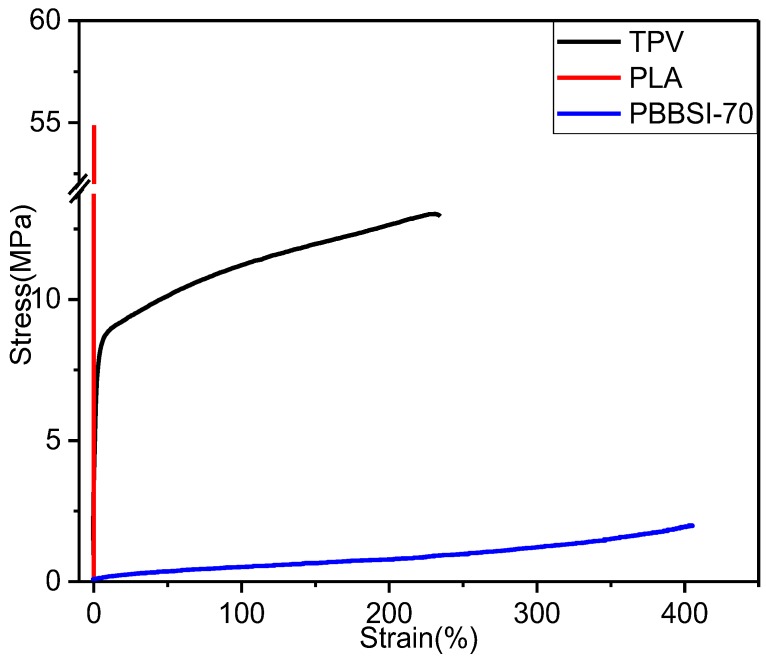
Stress–strain curves of the PBBSI/PLA TPV and its individual components.

The study of the mechanical properties of the PBBSI/PLA TPV is shown in Figure 9. The curve of neat PLA shows that it broke at only 6% strain, with high tensile strength as a typical brittle plastic behavior. Neat PBBSI broke at nearly 400% strain with low tensile strength, an indication of elastic features. However, the stress–strain curve of PBBSI/PLA was different with PLA and PBBSI; the TPV showed a tensile strength of 13.03 MPa and elongation at break of 231.32%. The stress–strain curve of the PBBSI/PLA TPV was between that of PBBSI and PLA, presenting its combined features of elastomer and plastic.

### 3.7. Thermomechanical Properties of the PBBSI/PLA TPV

**Figure 10:**
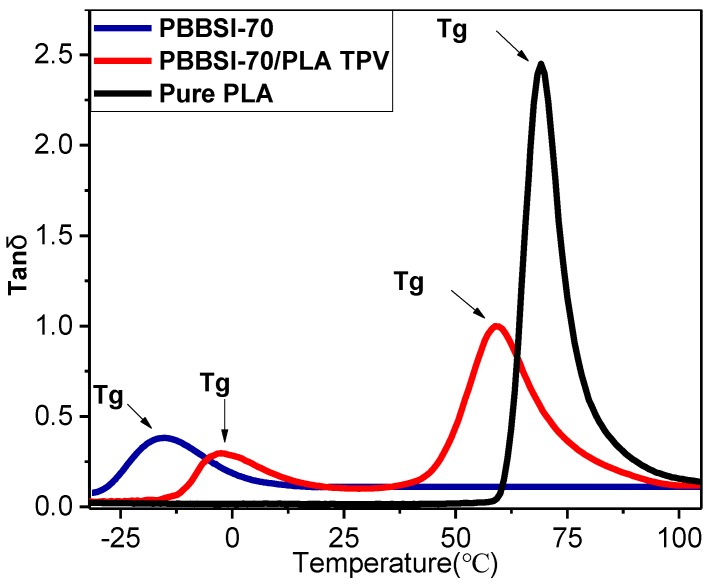
Thermomechanical curves of the PBBSI/PLA TPV and its individual components.

Figure 10 shows the thermomechanical properties of the PBBSI/PLA TPV measured by DMA by varying the measurement temperature. From the figure, the glass transition temperature of PBBSI-70 is at −15.4 °C while that of pure PLA is 68.8 °C; the PBBSI/PLA TPV has two glass transition temperatures, at −2.4 and 59.0 °C. The glass transition temperatures of the TPV were between the *T*_g_s of the pure PLA and PBBSI, and the two glass transition temperatures stayed close to each other, meaning that the two phases in the mixture had a good compatibility, guaranteeing the state of phase separation.

### 3.8. Rheological Properties of the PBBSI/PLA TPV 

**Figure 11:**
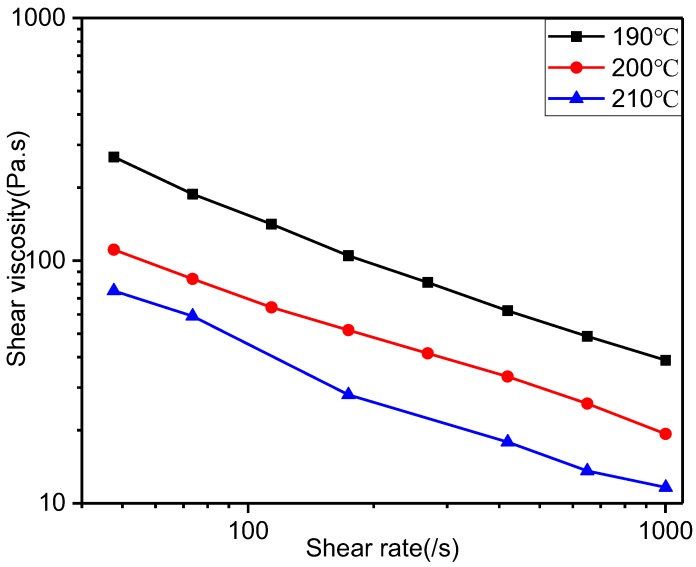
Rheological curves of the PBBSI/PLA TPV.

The study of the rheological properties of polymers is the basis for solving various problems in polymer processing, and as such, the PBBSI/PLA TPV curves of shear rate and shear viscosity are shown in Figure 11. The shear viscosity obviously decreased as the shear rate rose, indicating that the PBBSI/PLA was a non-Newtonian fluid with shear thinning. Furthermore, we changed the temperature to investigate the relationship between temperature and the shear viscosity, which can play a guiding role in processing. At the same shear rate, the shear viscosity decreased obviously with an increase of temperature. However, the PBBSI/PLA TPV showed unsteady flow in the course of shear thinning at 210 °C, which was mainly due to melt-burst of the PBBSI/PLA TPV. Therefore, the processing temperature of the PBBSI/PLA TPV should be below 210 °C. Furthermore, we measured the melting index of the PBBSI/PLA TPV by using a melt-flow indexer. Its melt index was 32.8 g/min, showing its excellent rheological property for processing.

### 3.9. 3D-Printing Study of the PBBSI/PLA TPV

**Figure 12:**
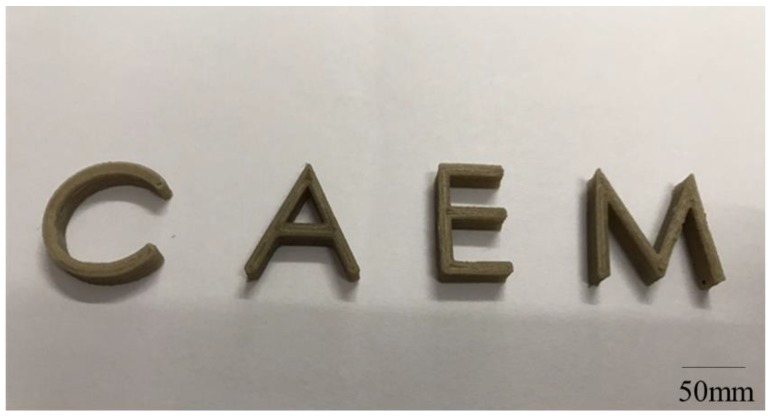

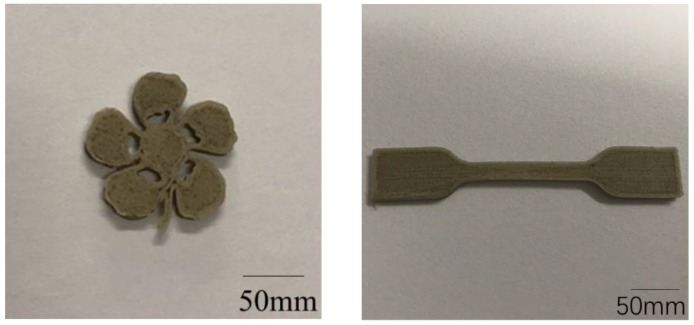
3D-printed samples of the PBBSI/PLA TPV.

Nowadays, with its rapid development, 3D-printing technology has become the focus of research on rapid formation (RP) technology. Both machinery and materials are highly needed to promote the progress of 3D-printing technology. At present, FDM printing is the most popular method, and it uses thermoplastic polymers as printing materials. We have prepared this novel TPV material with appropriate hardness and high fluidity on the basis of PLA and PBBSI. It not only meets the flowability requirement of FDM printing, but also provides high elasticity, which will enrich the options of FDM printing. There are several typical print products shown in Figure 12.

## 4. Conclusions

This study prepared a bio-based thermoplastic vulcanizate (TPV) using PLA and PBBSI copolyesters, which consisted of 2,3-butanediol, 1,4-butanediol, succinic acid and itaconic acid. The number-average molecular weights and polydispersities of the PBBSI copolyesters ranged from 20,000–40,000 g/mol and 3.2–5.0, respectively. The chemical structure and characteristic groups were characterized by FTIR and ^1^H NMR spectroscopy, and the thermal properties of the PBBSI copolyester were confirmed by TGA and DSC. Through the dynamic vulcanization process, the bio-based TPV was successfully prepared based on PLA and PBBSI elastomers. The PBBSI/PLA TPV has a good flowing property and high toughness for 3D printing.

## Figures and Tables

**Table 1:** Molecular weights of PBBSI copolyesters.

Molecular Weights					
	PBBSI-50	PBBSI-60	PBBSI-70	PBBSI-80	PBBSI-90
M_n_	39,944	26,174	25,376	23,396	21,907
PDI	4.5	5.0	3.7	3.2	4.7

**Table 2:** Compositions and molecular weights of PBBSI copolyesters.

Molar Compositions 1,4-BDO/2,3-BDO/SuA/IA		
Samples	Feed ^a^	Copolyester ^b^
PBBSI-50	0.25/0.25/0.45/0.05	0.27/0.23/0.48/0.02
PBBSI-60	0.20/0.30/0.45/0.05	0.22/0.27/0.49/0.02
PBBSI-70	0.15/0.35/0.45/0.05	0.18/0.32/0.48/0.02
PBBSI-80	0.10/0.40/0.45/0.05	0.13/0.35/0.50/0.02
PBBSI-90	0.05/0.45/0.45/0.05	0.09/0.40/0.49/0.02

^a^ Molar ratio in the initial feed; ^b^ molar ratio in the copolyester, determined by ^1^H NMR.

**Table 3:** Thermal properties of PBBSI polyesters.

Samples	PBBSI-50	PBBSI-60	PBBSI-70	PBBSI-80	PBBSI-90
*T*_d_,_10%_^a^ (°C)	398.5	396.6	394.9	394.1	382.8
*T*_d,max_^b^ (°C)	377.3	375.9	374.9	372.8	366

^a^ Temperature at which a 5% weight loss was observed in the TGA traces recorded at 10 °C/min; ^b^ temperature of maximum degradation rate.

## Supplementary Materials

The following are available online at www.mdpi.com/2073-4360/9/12/694/s1, Figure S1: Partial enlarged detail of ^1^H NMR spectra of PBBSI-70 copolyesters, Table S1: Mechanical properties of crosslinked PBBSI copolyesters.
