# Supplementary material for: Preparation and Properties of Novel Thermoplastic Vulcanizate Based on Bio-Based Polyester/Polylactic Acid, and Its Application in 3D Printing
Source: Polymers (Basel). 2017 Dec 9;9(12):694. doi: 10.3390/polym9120694 (PMC6418593; doi:10.3390/polym9120694)
Supplement: Supplementary file 1 [file polymers-09-00694-s001.pdf]

Electronic Supplementary Material (ESI) for Polymers.

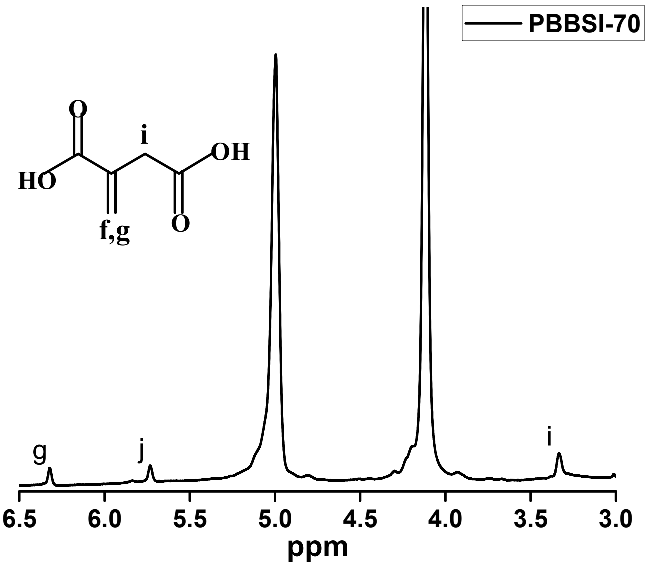

Fig S1 Partial enlarged detail of ^{1}H-NMR spectra of PBBSI-70 copolyesters

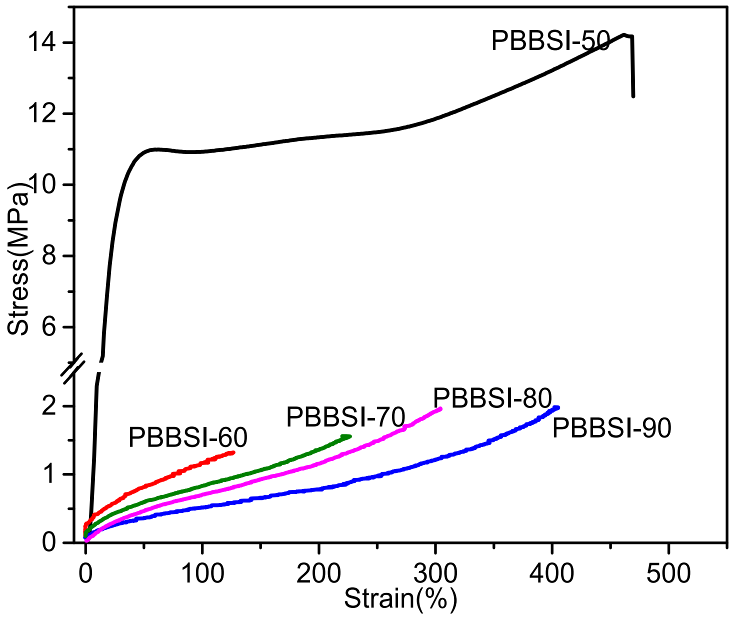

Fig S2 Stress-Strain curves of PBBSI copolyesters.

Table S1 Mechanical properties of crosslinked PBBSI copolyesters

sample	Elastic modulus	Max stress (Mpa)	Elongation at break (%)
PBBSI-50	37.4	14.3	467.8
PBBSI-60	——	1.3	125.9
PBBSI-70	——	1.6	223.5
PBBSI-80	——	1.9	304.2
PBBSI-90	——	2	405.4

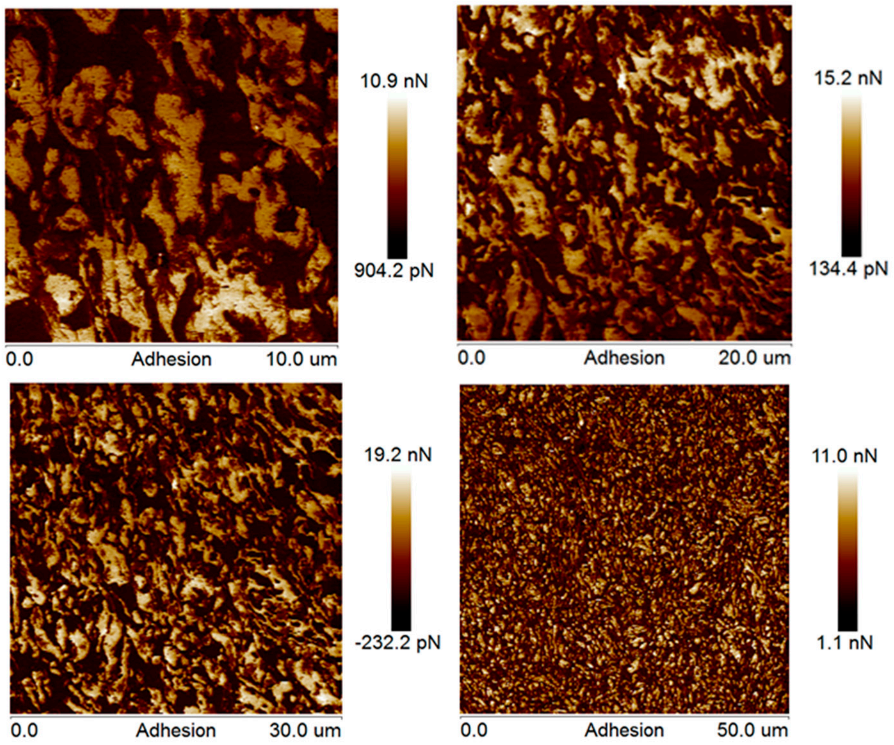

Fig S3 AFM micrograph PBBSI/PLA TPV((a)is 10μm; (b)is 30μm; (c)is 20μm; (d)is 50μm)
